# Allergic laryngitis: chronic laryngitis and allergic sensitization^☆^

**DOI:** 10.1016/j.bjorl.2019.02.001

**Published:** 2019-03-04

**Authors:** Andrea Campagnolo, Michael S. Benninger

**Affiliations:** a Universidade do Estado do Rio de Janeiro (UERJ), Rio de Janeiro, RJ, Brazil; b Otolaryngology Clinic, Rio de Janeiro, RJ, Brazil; c The Cleveland Clinic, Head and Neck Institute, Cleveland, United States

Allergic inflammation may affect both the upper and lower airways^1^ and allergic diseases may have a significant negative impact on the quality of life and the individual's productivity.^2^, ^3^, ^4^ Allergic rhinitis affects at least 20% of the American population^5^ and the prevalence rates are increasing. The relationship between upper and lower airway inflammatory diseases is increasingly recognized and has been described as a unified airway.^1^, ^6^ The concept of a unified airway is described as an inflammatory alteration in one part of the airway that causes inflammatory responses in other segments of the airway.^1^, ^6^, ^7^ Although the unified airway is well studied and described, the relationship of allergic disease and laryngeal symptoms and the role of allergy in chronic laryngitis is still poorly described and controversial.^8^ Recent studies have proposed that allergy may cause dysphonia by direct inflammation, trafficking of mucus through the upper or lower airway larynx, and compensatory behaviors such as cough that causes laryngeal edema.^9^

Laryngeal symptoms resulting from allergic laryngitis are not specific and include hoarseness, throat clearing, coughing and globus sensation.^3^ Although no specific laryngoscopic signs are pathognomonic for allergic laryngitis, findings associated with allergic laryngitis include dense endolaryngeal mucus, hyperemia and vocal fold edema.^10^ These signs and symptoms are also common in patients with laryngopharyngeal reflux (LPR) and therefore some studies discuss the possibility of allergic laryngitis being misdiagnosed as LPR.^10^, ^11^, ^12^, ^13^

Individuals with allergic rhinitis have a higher prevalence of dysphonia than non-allergic individuals.^9^, ^14^, ^15^ Singers with vocal symptoms are 15%–25% more likely to have allergic rhinitis than those without vocal symptoms.^16^ Simberg et al.^17^ evaluated college students with and without allergy and found that students with allergy reported significantly more vocal symptoms than those without allergy. The diagnosis of allergic laryngitis may be challenging. The symptoms of allergic laryngitis are not specific, there is the possibility of allergic laryngitis coexisting with LPR or asthma where the effects of coughing, increased mucus viscosity and the use of pulmonary inhaled medications all can play a role in the difficulty in isolating allergic laryngitis.^18^ Despite the suspected role of allergic inflammation causing chronic laryngitis, the term “allergic laryngitis” is still controversial.

What is the role of the larynx in the Unified Airway? According to Krouse,^6^ the respiratory tract, from the Eustachian tube, paranasal sinuses to the distal bronchioles function as a unified and interrelated unit. The larynx is located between the upper and lower airway, the mucus passes through the larynx descending the upper airway or ascending the lower airway. The mucosa of the larynx is similar to that of the rest of the respiratory tree and therefore it would be difficult to assume upper and lower airway allergic inflammation sparing the larynx.

Allergic laryngitis results from exposure to an inhaled allergen, causing symptoms of coughing and dysphonia and likely occurs through 3 mechanisms^6^, ^19^: (1) local inflammation of the larynx, nose or paranasal sinuses produces a system of upregulation of inflammatory mediators that pass through the circulation and increase the production of local mucus, (2) trafficking of mucus through the larynx and (3) edema of the mucosa resulting from compensatory mechanisms such as throat clearing and coughing. According to the concept of a unified airway, allergic laryngitis would result from a systemic spread of local inflammation involving the entire respiratory tract.^19^, ^20^

Clinical symptoms of allergic laryngitis include frequent symptoms of any chronic laryngitis such as coughing, throat clearing, foreign body sensation, excessive mucus in the larynx, post nasal drainage and occasional dysphonia. These symptoms are not specific and are common in patients with LPR, often leading to misdiagnosis of allergic laryngitis such as LPR.^10^, ^18^ These symptoms are also present in patients with acute upper respiratory tract infections and in chronic, non-allergic rhinosinusitis.

Most patients with vocal disorders resulting from chronic laryngitis present with various symptoms that are present in different inflammatory conditions, making it a challenge to define the cause of the symptoms, since more than one cause can coexist.^18^ Asthma and its treatment may cause dysphonia, and use of medications that cause dryness such as antihistamines, decongestants and pulmonary inhalers, can all cause laryngeal symptoms.^9^ The symptoms of allergic laryngitis are therefore non-specific and include hoarseness, throat clearing, globus sensation, excessive mucus, sore throat and the sensation of a post nasal drip. As mentioned above, these symptoms are common to other inflammatory disorders and recent studies have discussed the possibility of an overdiagnosis of LPR and an underdiagnosis of allergic laryngitis.^8^, ^10^, ^11^, ^13^

Despite some controversies regarding RFL, which is defined as a retrograde flow of gastric contents to the larynx and pharynx, coming in contact with tissues of the upper aero-digestive tract,^21^ 24 h pH monitoring with two probes and multichannel intraluminal impedance and manometry are considered the gold standard in the diagnosis of reflux and LPR. These tests however are not used routinely because of patient discomfort and cost.^21^, ^22^ More commonly, the diagnosis is made based on clinical symptoms suggesting reflux, the response to an empirical behavioral and drug treatments and endoscopic findings of mucosal changes.^23^

Belafsky et al.,^24^ developed a patient-based questionnaire to evaluate the symptoms related to LPR, the Reflux Symptom Index (RSI), and also a scale to rating the findings of laryngoscopy to predict the presence of LPR, the Reflux Finding Score.^25^ Due to the subjectivity of the results of these evaluations, the low specificity and inter-rater reliability, these scales are not routinely used in clinical practice.^18^, ^26^, ^27^ However, in the study by Erdem et al.,^10^ they found a high inter-rater reliability for thick laryngeal mucus as an allergy predictor.

Brook et al.^8^ demonstrated high positivity in the *in vitro* allergy test in patients with chronic laryngitis symptoms, similarly to patients with rhinitis and sinusitis, diseases most associated with allergy. In the study of Randhawa et al.^13^ patients with dysphonia had a higher incidence of allergy, diagnosed by skin prick test (SPT) compared to LPR, diagnosed by RSI and RFS. All patients with LPR presented concomitant allergy. In a subsequent study, Randhawa et al.,^28^ found that the degree of allergy of allergic patients correlated with the severity of vocal symptoms assessed by the Voice Handicap Index Score (VHI).

In the study by Koc et al.^29^ acoustic and stroboscopic findings of the larynx and VHI questionnaire scores were investigated in 30 patients with allergic rhinitis compared to 30 controls without age-and-sex-matched allergic rhinitis. No difference was observed between patients with allergic rhinitis and the control group in relation to stroboscopic findings, but the values of VHI and S/Z ratio (the length of time a person can sustain the sound ‘s’, the length of time they can sustain the sound ‘z’), which is often increased in laryngeal pathologies, were significantly higher in the allergic rhinitis group, suggesting a relationship between allergy and dysphonia.

Millquvist et al.^29^ also evaluated 30 allergic patients and 30 non-allergic controls using the VHI questionnaire to assess vocal disability. During the allergic seasonal period, allergic patients had a significant increase in respiratory and vocal symptoms compared to non-allergic controls. Krouse et al.^30^ evaluated stroboscopy and VHI scores in subjects who were allergic to dust mites (as diagnosed by SPT) compared to non-allergic individuals. Allergic subjects presented significantly higher VHI scores but no differences were observed between the groups in appearance or laryngeal function. The review study by Garret et al.^31^ reported that empiric treatment for LPR is widely used by otolaryngologists and clinicians in patients with non-specific symptoms of chronic laryngitis. This study emphasizes the importance of making the differential diagnosis with allergic laryngitis, asthma and even muscular tension dysphonia (TMD) to avoid unnecessary treatments and delays in the correct diagnosis.

The causal relationship between the direct introduction of the allergen and the appearance of laryngopharyngeal symptoms has been investigated. Reidy,^32^ Dworkin and colleagues^33^ performed two studies to investigate these relationships. In the first,^32^ they developed trans-oral challenge using antigen dust mites nebulized (1:200) and placebo in sensitized patients. There was no significant difference between the nebulized patients with mites and those with placebo in the vocal analyses, videostroboscopy and VHI. In the second study,^33^ in a randomized, placebo-controlled, double-blind study, the authors introduced low (1:100) and high (1:40) concentrations of dust mites in sensitized patients. The study was prematurely suspended after 2 patients developed vocal edema, increased secretions, dysphonia, cough, and respiratory dysfunction. No reaction occurred on exposure with antigen at low concentration and on 1 control that completed the study.

Roth et al.^34^ conducted a prospective, double-blind, placebo-controlled study in which subjects served as controls. Transoral inhalants were used in 5 patients with no evidence of lower airway reaction to methacholine challenge. All patients presented an increase in the phonatory pressure threshold (PTP) when compared to placebo inhalation. In a more recent study, Belafsky and colleagues^35^ used an experimental animal model for chronic laryngitis. Indian pigs were sensitized with house dust mite allergen (HDMA) and exposed to them alone and also associated with iron soot for 6 weeks. The combination of iron soot with house dust mite allergen (HDMA) caused submucosal and epithelial eosinophilia in the glottis, subglottis and trachea. Finally, Silva Merea and colleagues^4^ have investigated a large cohort of 879 *in vitro* positive allergic patients and found that 9.8% of these patients had simultaneous allergic diagnoses. Of these, 78% had dysphonia, 21% with non-infectious laryngitis and 15% with a globus sensation. When combining allergens into categories, dust mite sensitization was the most common (50%) closely followed by grass and animal dander (49% each).

As demonstrated in this review, several researchers have sought to find a relationship between the symptoms of chronic laryngitis and allergic sensitization. Despite the evidence found by these researchers, the pathogenesis of this relationship is not yet clearly defined. Laryngeal symptoms and signs attributed to allergic laryngitis are non-specific and overlap with other diseases, mainly LPR. Most authors report that the presence of dense endolaryngeal mucus should alert for the presence of allergic laryngitis. Some researchers have shown that the introduction of allergens directly into the larynx causes physical and functional changes in the larynx. Allergic laryngitis has also been associated with worsening of vocal quality (increase in VHI score) and allergy treatment is associated with improvement of these indexes.

This review suggests that allergic sensitization should be considered in the differential diagnosis of patients with symptoms of chronic laryngitis, and LPR should not be the only diagnosis considered by the otorhinolaryngologist or clinical evaluation. Randomized clinical prospective studies are needed to establish more clearly the association of allergic disease with laryngeal symptoms. With better understanding of the role of allergic inflammation in the larynx and the most effective treatments management guidelines of allergic laryngitis can be developed.

## Conflicts of interest

The authors declare no conflicts of interest.
